# A Y374X TDP43 truncation leads to an altered metabolic profile in amyotrophic lateral sclerosis fibroblasts driven by pyruvate and TCA cycle intermediate alterations

**DOI:** 10.3389/fnagi.2023.1151848

**Published:** 2023-05-11

**Authors:** Scott P. Allen, Afnan Al Sultan, Elaine Kabucho Kibirige, Erin Tonkiss, Keaton J. Hamer, Lydia M. Castelli, Ya-Hui Lin, Sarah Roscoe, Nikolaos Stefanidis, Richard J. Mead, J. Robin Highley, Johnathan Cooper-Knock, Guillaume M. Hautbergue, Paul R. Heath, Janine Kirby, Pamela J. Shaw

**Affiliations:** Sheffield Institute for Translational Neuroscience (SITraN), University of Sheffield, Sheffield, United Kingdom

**Keywords:** ALS, fibroblasts, metabolism, pyruvate, TCA, transcriptomics, metabolomics

## Abstract

A p.Y374X truncation in TARDBP was recently shown to reduce expression of TDP43 in fibroblasts isolated from ALS cases. In this follow up study focused on assessing the downstream phenotypic consequences of loss of TDP43 in the context of the truncation, we have shown a striking effect on the fibroblast metabolic profile. Phenotypic metabolic screening uncovered a distinct metabolic profile in TDP43-Y374X fibroblasts compared to controls, which was driven by alterations in key metabolic checkpoint intermediates including pyruvate, alpha-ketoglutarate and succinate. These metabolic alterations were confirmed using transcriptomics and bioenergetic flux analysis. These data suggest that TDP43 truncation directly compromises glycolytic and mitochondrial function, identifying potential therapeutic targets for mitigating the effects of TDP43-Y374X truncation.

## Introduction

Amyotrophic lateral sclerosis (ALS), is a neurological disorder resulting in degeneration of both upper and lower motor neurons, resulting in the progressive failure of the neuromuscular system. Death typically occurs 2-3 years post symptom onset, due to a lack of effective therapies (McDermott and Shaw, 2008). Up to 10% of ALS cases are familial, with multiple genes identified including mutations in the *TARDBP* gene which encodes TDP43 (Renton et al., 2014). Interestingly, ubiquitylated neuronal and glial cytoplasmic inclusions containing TDP43, are a neuropathological hallmark of ALS irrespective of a mutation being present in the protein (Neumann et al., 2006; Sreedharan et al., 2008). Mitochondrial and metabolic dysfunction are observed early in ALS pathophysiology and may influence disease progression rates due to the metabolic and catabolic pathways being susceptible to the disease process (Haeusler et al., 2016; Tefera and Borges, 2016; Vos et al., 2017; Vandoorne et al., 2018). Lipid peroxidation, mitochondrial depolarisation and uncoupling linked to increased reactive oxygen species production, can disrupt the electron transport chain (ETC), calcium buffering and ATP generation. This has been observed both in the CNS and in peripheral models of disease (Ferraiuolo et al., 2011; Bartolome et al., 2013; Allen et al., 2014, 2015, 2019a,b; Raman et al., 2015; Tefera and Borges, 2016; Valbuena et al., 2016; Konrad et al., 2017).

There is a wealth of evidence linking TDP43 mutation and metabolic dysfunction (for a recent in depth overview see Floare and Allen, 2020). In the context of familial TDP43 associated ALS, it was recently noted that mutant TDP43 causes complex I disassembly by preferentially binding and impairing the processing of ND3 and ND6 mRNAs (Wang et al., 2016). Moreover, TDP43 mutations have been associated with increased ROS, impaired mitophagy, altered mitochondrial morphology and disrupted mitochondrial-ER interactions (Hong et al., 2012; Stoica et al., 2014). With this is mind, intrinsic metabolic flexibility allowing cells to either switch between energy generating pathways, or to upregulate catabolic pathways to meet energy demands may be protective. Recently, upregulation of the glycolytic rate-limiting enzyme PFK-1 was reported in a *Drosophila model* of TDP43-ALS, which was recapitulated in patient derived iPSC motor neurons with TDP43 pathology (Manzo et al., 2018). GLUT-3 overexpression in fly neurons or glia, significantly improved locomotor defects and improved synaptic vesicle recycling. Furthermore, a high sugar diet mitigated against TDP43 associated reduction in lifespan.

If metabolic manipulation is beneficial in the context of TDP43-ALS, then a greater understanding of how familial TDP43 mutations affect the catabolic pathways in human derived material is imperative. In a recent study, we reported a multi-generational ALS pedigree with a TDP43 Y374X truncation in the glycine-rich, C-terminal domain of the protein (Cooper-Knock et al., 2022). As previously stated, there is significant published work on the link between TDP43 point mutations and metabolic dysfunction. However, to the best of our knowledge, the effect of TDP43 truncation on metabolic function has not been previously studied and could uncover novel mechanisms of motor neuron injury. As reported in two recent studies (Allen et al., 2019a,b), we established a phenotypic metabolic screening approach in fibroblasts from C9orf72 and sporadic ALS cases as well as induced neuronal progenitor cell (iNPC) iAstrocytes derived by reprogramming the fibroblasts using a modified Yamanaka approach (Meyer et al., 2014). Fibroblasts are useful initial tool to investigate pathogenic dysfunction in human derived material and in many cases recapitulate the defects observed in the CNS (Allen et al., 2014, 2015; Raman et al., 2015; Onesto et al., 2016; Konrad et al., 2017; Veyrat-Durebex et al., 2019). Therefore, we employed this technique in fibroblasts isolated from our TDP43 truncation pedigree and validated the findings using transcriptomics, metabolic flux analysis and protein expression analysis in postmortem human motor cortex samples (Cooper-Knock et al., 2022).

## Materials and methods

All chemicals were purchased from Sigma unless otherwise stated.

### Ethical approval

Informed consent was obtained from all participants before skin sample collection (Study number STH16573, Research Ethics Committee reference 12/YH/0330). Autopsy tissues were donated to the Sheffield Brain Tissue Bank (SBTB) with appropriate consent. The SBTB Management Board gave ethical approval for this study under the provision to act as a Research Tissue Bank as approved by the Scotland A Research Ethics Committee (Ref. 08/MRE00/103).

### Human biosamples

Experiments were carried out using samples obtained from three TDP43 truncation ALS cases (c.1119_1120delTT, p.Y374X, and six matched controls (Table 1). The average age at time of skin biopsy in the ALS cases was 53.67 (standard deviation 14.01) years and 52.67 (standard deviation 11.71) years in controls (three females, three males).

**Table 1:** Fibroblast lines used for metabolic profiling.

**ID**	**Age at biopsy**	**Sex**	**TDP status**
Control 209	67	F	NA
Control 3050	66	M	NA
Control 2303	51	M	NA
Control 153	51	F	NA
Control 173	41	F	NA
Control 155	40	M	NA
Patient 194	68	M	Y374X
Patient 193	53	M	Y374X
Patient 192	40	M	Y374X

### Human fibroblast cultures

In accordance with local ethics committee guidelines, forearm skin biopsies were obtained from all subjects after informed consent. Fibroblast cell cultures were established in minimal cell culture medium (Lonza) supplemented with 10% fetal calf serum (Labtech), 2 mM glutamine (Lonza), 50 mg/ml uridine, vitamins, amino acids and 1mM sodium pyruvate (Gibco) in humid incubators at 37°C with 5% CO_2_.

### Phenotypic metabolic profiling

Phenotypic metabolic profiling was performed as previously described (Allen et al., 2019a,b). Briefly, fibroblasts were plated at 16,000 cells per well in fibronectin coated half area size 96 well plates (Greiner). Fibroblasts were incubated in 50 μl IFM-1 media (Biolog) in the presence of the metabolites from the PM-M1 plates (Biolog) and then incubated at 37°C/5% CO_2_ for 40 h. Subsequently, 10 μl of redox dye mix MA (Biolog) was added to each well and the plates sealed. Dye color change was measured for 300 min using a BMG Omega Pherastar at 590/790 nM (790 nM was removed from 590 nM to account for background values). After incubation, the plates were washed three times with 100 μl PBS and stored overnight before cell counting. All results were normalized to cell number by addition of CyQUANT (Invitrogen) to each well as per the manufacturer's instructions. Principle component analysis (PCA) plots were generated using Qlucore Omics Explorer 3.6, with *p* ≤ 0.05 taken as significant. Qlucore determines eigenvectors (also known as principal components), which calculate the directions of a feature in space. The software orders the eigenvectors based on the level of the total variance captured by each component, taking into account all variables or samples. The eigenvector values govern the magnitude of separation and the data variation along each specific axis. For all kinetic analysis a two-way ANOVA with a Sidak post-test analysis was performed.

### Metabolic flux analysis

Mitochondrial and glycolytic stress test analysis in fibroblasts were performed on an XF24 Seahorse bioanalyser as previously described (Allen et al., 2014, 2015, 2019a; Raman et al., 2015). Specifically for metabolic substitution assays, fibroblast media was supplemented with 0.3 mM glutamine and 5 mM glucose or either 5 mM pyruvic acid, butyric acid, alpha ketoglutaric acid, succinamic acid or adenosine (Sigma) for 40 h prior to metabolic flux analysis. Flux analysis was performed using XF basal media (Agilent) supplemented with the combination of metabolic substrates listed above. Metabolic flux analysis under physiological and stress conditions were assessed following sequential addition of the mitochondrial inhibitors oligomycin, FCCP and antimycin/rotenone (Sigma) as previously described (Allen et al., 2014, 2015). Flux data were normalized to CyQUANT fluorescence in each well as a proxy for cell number following the manufacturer's instructions as previously described (Allen et al., 2019a).

### Fibroblast transcriptomic analysis

Total RNA was extracted from pelleted fibroblasts using the Trizol method (Invitrogen), following the manufacturer's instructions. The RNA quality and quantity were established using the Agilent Bioanalyser (Agilent) and the Nanodrop 1000 (Thermo Fisher). Transcriptomic analysis was then carried out to compare the gene expression profile of RNA derived from the samples of the TDP43 mutant fibroblasts compared to the controls using the HTA 2 GeneChip^®^ microarrays from Affymetrix. 200ng of total RNA in 3 μl RNase free water was prepared for hybridization to the HTA 2 arrays using the *GeneChip*^®^ *WT PLUS* kit from Affymetrix following manufacturer's instructions. Briefly the RNA was combined with diluted poly A controls as labeling controls and reverse transcribed. The resultant cDNA was converted to double stranded DNA and an *in vitro* transcription reaction was used to generate cRNA. A second reverse transcription generated a further RNA:DNA hybrid. The RNA was enzymatically digested, and the ssDNA molecule fragmented and labeled with Biotin. A hybridization solution was prepared including pre-labeled hybridization controls and the appropriate mixture applied to a microarray chip. The arrays were hybridized for 16 hours at 45°C with 60 rpm followed by washing and staining using the Affymetrix wash/stain/label kit and scanned on the Affymetrix Gene Chip 3000 7G scanner. The CEL files were subject to quality control checks using Expression Console v1.0 software (Affymetrix). Further data analysis was carried out using the Qlucore Omics Explorer software (Qlucore) to establish the differentially expressed gene list used for downstream comparisons. The *p*-value was set to < 0.05 and fold change to ≥± 1.5.

### Neuropathology

One cerebral hemisphere, half the midbrain and brainstem, a portion of the cerebellum, and segments of the spinal cord from cervical, thoracic and lumbar levels were donated from five controls and one TDP43 case (Cooper-Knock et al., 2022) to the Sheffield Brain Tissue Bank after consent from the next of kin. Brain and spinal cord samples were snap-frozen at autopsy using liquid nitrogen. The remaining central nervous system tissue was processed as previously described (Cooper-Knock et al., 2022). Motor cortex tissue (~0.1 g) was lysed and homogenized (Precellys evolution homogenizer and 8 × 1.4 mm Zirconium Oxide beads) in 400 μl RNase free RIPA lysis buffer (2% SDS, 50 mM Tris, pH 8.0, 150 mM NaCl, 1% IGEPAL CA-630 0.5% sodium deoxycholate, benzonase (250 units/10 ml), protease inhibitor cocktail, 2 mM PMSF) at 5,500 rpm for 2 × 30 s, followed by 10-min incubation on ice and one further homogenisation step. Cells lysates were collected after centrifugation at 17,000 *g* for 10 min at 4°C. Protein concentrations were determined by BCA analysis as per the manufacturer's instructions.

### Western blot analysis

30 μg was loaded onto a 10% SDS-PAGE Mini-PROTEAN Tetra Handcast systems (Bio-Rad). Proteins were resolved and transferred to a nitrocellulose membrane at 250 mA for 1 h. The membranes were incubated for 1 h with blocking solution containing 5% BSA in Tris-buffered saline with 0.01% Tween (TBS-T). Subsequently, membranes were incubated overnight at 4°C with the following primary antibodies, Pyruvate Carboxylase (PC GTX132002, GeneTex) 1/2,000, Mitochondrial Pyruvate Carrier 2 (MPC2-20049-1-AP, Proteintech) 1/2,000, dihydrolipoamide s-acetyltransferase (DLAT-13426-1-AP, Proteintech) 1/3,000, SUGT succinyl-coA:glutarate-coA transferase (SUGCT-21589-1-AP, Proteintech) 1/1,000, dihydrolipoamide dehydrogenase (DLD- sc-365977, SantaCruz) 1/500, alpha-glucosidase (GAA- sc-373745, SantaCruz) 1/1,000 Beta-actin (20536-1-AP, Proteintech) 1/3,000 in blocking solution. Membranes were washed in TBS-T prior to incubation with HRP-linked rabbit secondary antibody at 1 in 10,000 before detection by chemiluminescence (EZ-ECL HRP kit, Biological Industries) using a Licor Odyssey Fc). Protein quantification levels were obtained by densitometry using Image Studio Lite (Version 5.2.5) and normalization to the loading controls.

### Statistical analysis

All data were assessed for Gaussian distribution prior to either unpaired *t*-test with a Welch correction or Mann-Whitney analysis (if non-parametric) for two groups. For three or more groups, one way ANOVA with a Tukey post-test or Kruskal Wallis with Dunn's post-test (if non-parametric) were performed. The metabolic profiling data were analyzed as described above. All graphs were generated showing standard deviation using Graphpad Prism version 8.4.3 (GraphPad Software, La Jolla, CA, USA).

## Results

### Truncated TDP43 cases have an altered metabolic profile compared to age and sex matched controls

**Figure 1:**
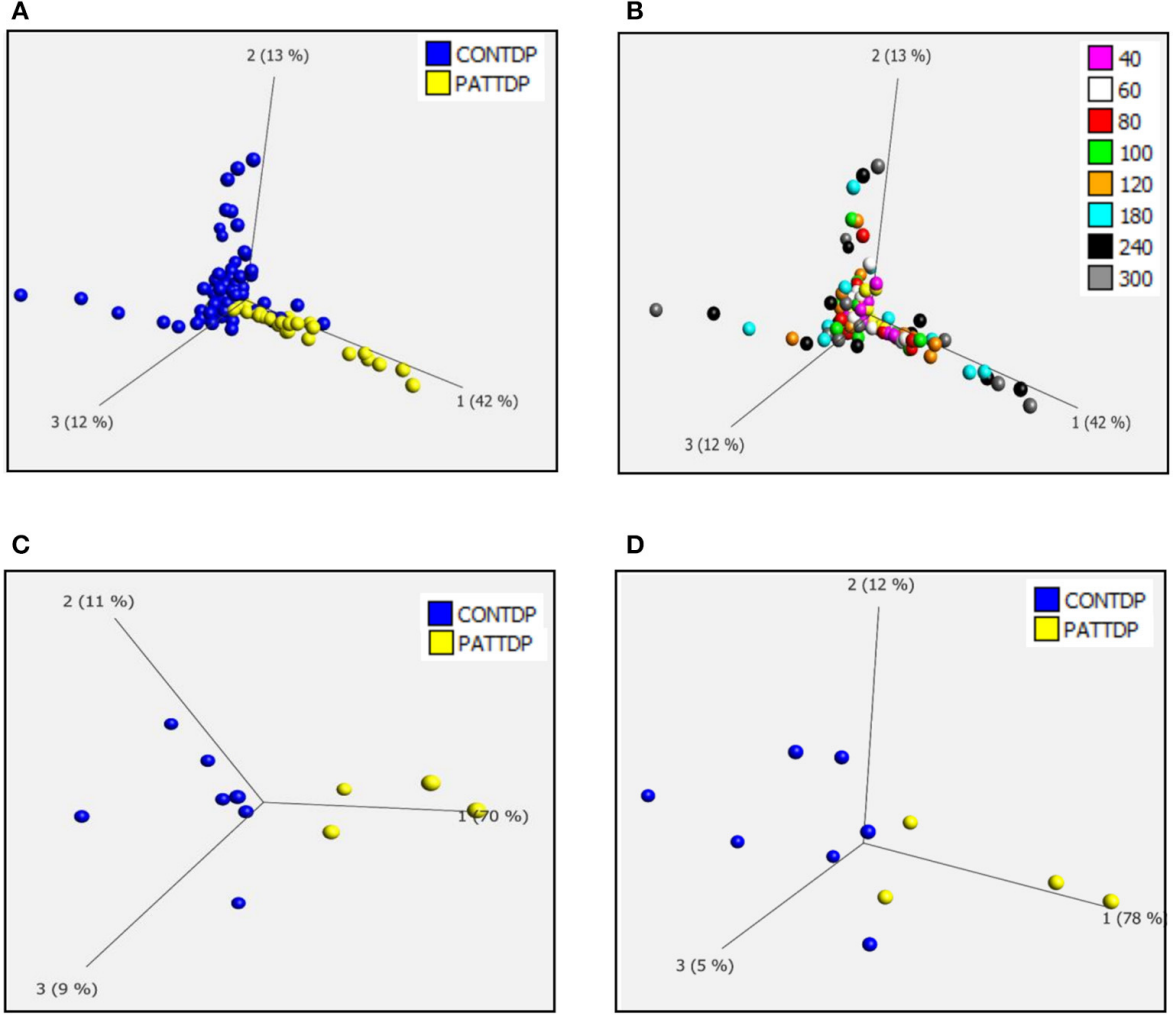
Fibroblasts derived from truncated TDP43 cases have an altered metabolic profile. **(A)** Principal component analysis of control fibroblasts (blue, Con) and TDP fibroblasts (yellow Pat) at phenotypic metabolic profiling time points. **(B)** Principal component analysis of fibroblasts colored for individual time points between 20 and 300 min. **(C)** PCA of control fibroblasts (blue, Con) and TDP43 fibroblasts (yellow PATTDP) at 120 min. **(D)** PCA of control fibroblasts (blue, Con) and TDP43 fibroblasts (yellow, PATTDP) at 240 min. Data are presented as mean of three biological replicates using six control fibroblasts and three TDP43 truncation fibroblasts. Analysis performed on Qlucore with the *P*-value set to 0.05. Q-values were 0.103 for control fibroblasts vs. TDP43 fibroblasts. Percentage values represent eigenvectors calculated for each analysis. The higher the percentage the greater the confidence of the separation based on the vector.

As previously described (Allen et al., 2019a,b), we used phenotypic metabolic profiling to assess the effect of TDP43 truncation on the metabolic profile of fibroblasts. This methodology enables the comparison of ALS vs. healthy fibroblast cell models by measuring energy production rates from 91 energy substrates simultaneously in 96 well plates. Using a redox dye, the technology measures the ability of cells to produce NAD (P) H (nicotinamide adenine dinucleotides) in real time, via NADH producing catabolic pathways that utilize a range of metabolic substrates. This approach produces an unbiased measurement of metabolic function at over 90 different metabolic pathway points simultaneously. The advantage of this approach is that it enables a global metabolic profile to be measured at different time points after addition of the redox dye and concomitantly allows identification of the metabolic substrates that define the global profile. During the assay, taking all time points into account (40-300 minutes), there was significant overlap between the control and TDP43 fibroblasts (Figures 1A, B). However, better separation was observed between the two groups at later time points where the greatest variation in the data was observed (Figures 1C, D). Therefore, when we focused on individual time points later in the assay (120 min, Figure 1C and 240 min, Figure 1D), we observed greater separation especially at 120 min which is typically in the linear phase of the assay (Allen et al., 2019a,b). These data suggest that, as previously observed in C9orf72 fibroblasts and iAstrocytes, phenotypic metabolic profiling can be used to elucidate an altered metabolic profile in TDP43 models of ALS.

### TDP43 truncation causes reduction of metabolism of metabolic intermediates that feed into glycolysis and the tricarboxylic acid (TCA) cycle

**Figure 2:**
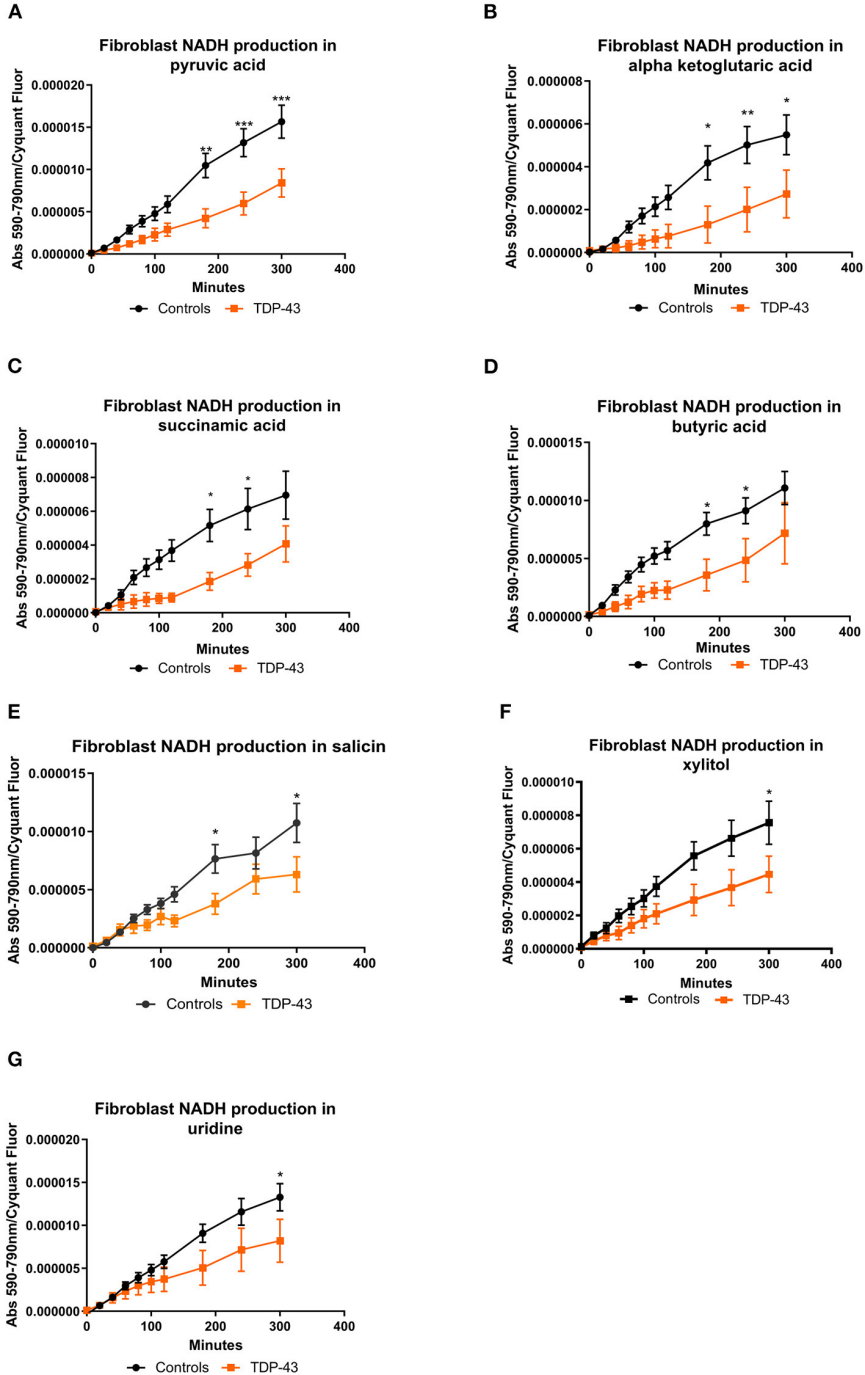
**(A)** NADH production in fibroblasts with pyruvic acid as the sole energy source. **(B)** NADH production in fibroblasts with ketoglutaric acid as the sole energy source. **(C)** NADH production in fibroblasts with succinamic acid as the sole energy source. **(D)** NADH production in fibroblasts with butyric acid as the sole energy source. **(E)** NADH production in fibroblasts with salicin as the sole energy source. **(F)** NADH production with xylitol as the sole energy source. **(G)** NADH production in fibroblasts with uridine as the sole energy source. Controls depicted in black and TDP43 in orange. Fibroblast NADH production was measured using a BMG Pherastar plate reader taking absorbance readings over a 6-h period. Data presented as mean with standard error for six controls and three TDP43 cases analyzed in triplicate. Background intensity values were subtracted from raw data values before being normalized to cell number (by Cyquant analysis). Two-way ANOVA, with Sidak post-test, was performed. *P ≤ 0.05, **P ≤ 0.01, ***P ≤ 0.001.

The fibroblast control lines metabolized 23 substrates within 95% of the glucose positive control. As with our previous studies (Allen et al., 2019a,b) the fibroblast lines were predominantly reliant on saccharides but were able to metabolize nucleosides and a selection of carboxylic acids (Supplementary Figure 1). When we focused in on those metabolites in our screen that were significantly altered from the PCA analysis we found that truncated TDP43 fibroblasts had reduced metabolism of energy substrates feeding the TCA cycle including pyruvic acid, alpha ketoglutaric acid, succinamic acid (as well as mono-methyl succinate (Supplementary Figure 2M) and butyric acid (Figures 2A–D). Furthermore, TDP43 truncated fibroblasts had reduced metabolism of the glucoside salicin, the sugar alcohol xylitol and the pyrimidine nucleoside uridine (Figures 2E–G). Overall, of the 23 metabolic substrates utilized, 15 showed no significant difference between controls and TDP43 cases (Supplementary Figure 2).

**Figure 3:**
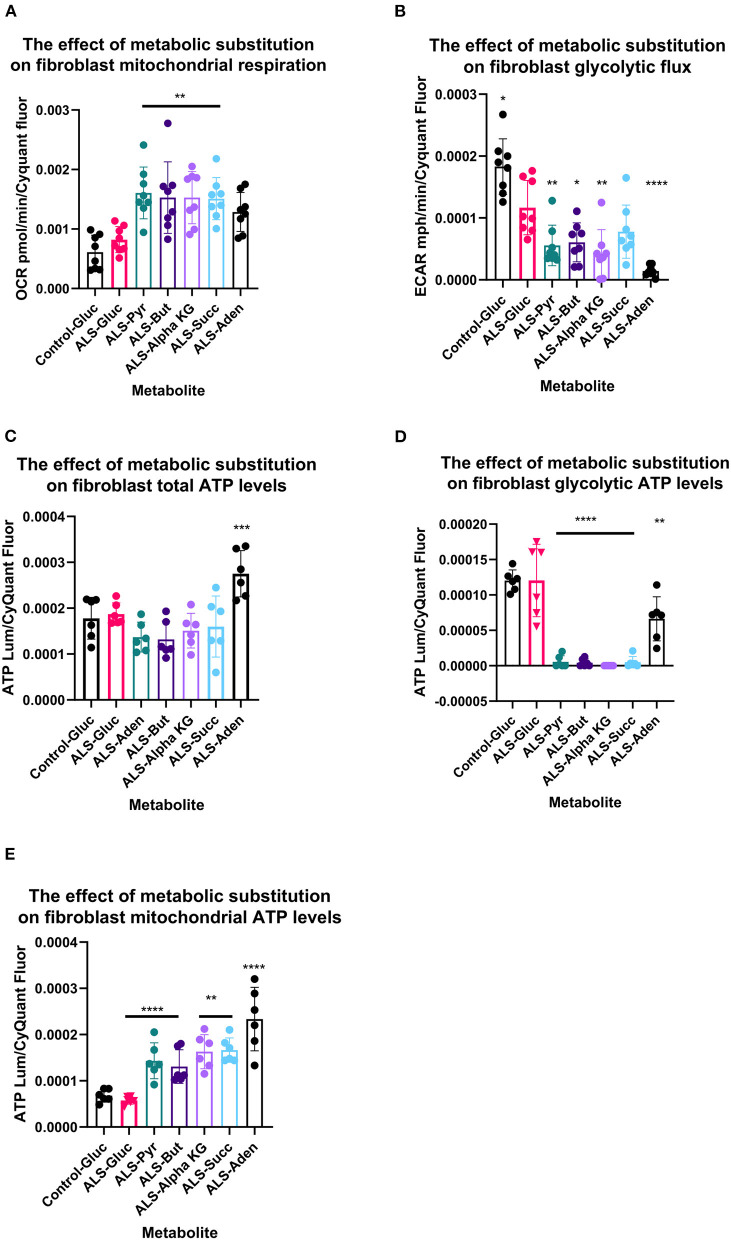
Metabolic substitution reduces glycolytic energy production and promotes mitochondrial energy production in TDP43 fibroblasts. **(A)** The effect of metabolic substitution on fibroblast mitochondrial function. **(B)** The effect of metabolic substitution on fibroblast glycolytic flux. **(C)** The effect of metabolic substitution on fibroblast ATP levels. **(D)** The effect of metabolic substitution on fibroblast glycolytic ATP levels. **(E)** The effect of metabolic substitution on fibroblast mitochondrial ATP levels. Data presented as mean with standard deviation of two control and two ALS cases performed *n* = 3/4 prior to one way ANOVA with Tukey's post-test analysis or Kruskal Wallis non-parametric analysis with Dunn's post-test analysis. *p ≤ 0.05, **p ≤ 0.01. ***p ≤ 0.001. ****p ≤ 0.0001. OCR = oxygen consumption rate. ECAR = extracellular acidification rate.

### TDP43 fibroblasts have alterations in genes that control carbon entry into the mitochondria

Transcriptomic analysis (for the full dataset see Supplementary Table 1) of the TDP43 cases compared to controls identified a number of genes involved in the metabolic processes identified in phenotypic screening. These included decreased expression of the mitochondrial membrane carriers including translocase of outer mitochondrial membrane 7 (TOMM7), solute carrier family 25 (mitochondrial carrier, phosphate carrier) member 23 (SLC25A23), and the mitochondrial pyruvate carrier (MPC2, Table 2). Furthermore we found extensive alterations in TCA cycle associated enzymes including SUGT (succinyl-CoA, glutarate-CoA transferase), DLD (dihydrolipoamide dehydrogenase) and ACSS3 (acyl-CoA synthetase short-chain family member 3. Moreover, we found alterations in a number of membrane associated transporters and receptors including loss of the uridine receptor P2RY13.

**Table 2:** Differentially expressed metabolic genes between fibroblasts from control and truncated TDP43 cases.

**ID**	**C Avg (log2)**	**T Avg (log2)**	**FC**	***P*-value**	**Gene symbol**	**Description**
TC07000255.hg.1	5.79	6.39	−1.51	0.0369	SUGCT	succinyl-CoA:glutarate-CoA transferase
TC07001190.hg.1	8.13	8.74	−1.52	0.0200	TOMM7	translocase of outer mitochondrial membrane 7 homolog
TC19001097.hg.1	7.2	7.86	−1.58	0.0129	SLC25A23	solute carrier family 25 (mitochondrial carrier; phosphate carrier), member 23
TC01003484.hg.1	7.47	8.20	−1.65	0.0071	MPC2	mitochondrial pyruvate carrier 2
TC03001906.hg.1	3.84	4.68	−1.79	0.0246	P2RY13	purinergic receptor P2Y, G-protein coupled, 13
TC06004141.hg.1	9.5	10.45	−1.94	0.0210	SOD2	superoxide dismutase 2, mitochondrial
TC17000915.hg.1	7.38	8.35	−1.97	0.0018	GAA	glucosidase, alpha; acid
TC05001270.hg.1	9.26	8.65	1.52	0.0256	NADK2	NAD kinase 2, mitochondrial
TC02002052.hg.1	10.84	10.23	1.53	0.015	IMMT	inner membrane protein, mitochondrial
TC01003543.hg.1	6.81	6.19	1.54	0.0169	MRPS14	mitochondrial ribosomal protein S14
TC01002942.hg.1	10.30	9.62	1.59	0.0095	SLC25A24	solute carrier family 25 (mitochondrial carrier; phosphate carrier), member 24
TC12000672.hg.1	6.58	5.87	1.64	0.0101	ACSS3	acyl-CoA synthetase short-chain family member 3
TC07000697.hg.1	10.74	9.98	1.69	0.0196	DLD	dihydrolipoamide dehydrogenase
TC02001859.hg.1	9.41	8.64	1.7	0.0205	MTIF2	mitochondrial translational initiation factor 2
TC03001787.hg.1	11.56	10.73	1.79	0.0081	MRPL3	mitochondrial ribosomal protein L3
TC06002142.hg.1	6.19	5.09	2.15	0.0218	MTFR2	mitochondrial fission regulator 2
TC01001990.hg.1	9.93	8.66	2.41	0.0398	COX20	COX20 cytochrome c oxidase assembly factor

FC, fold change.

### TDP43 fibroblasts display alterations in metabolic flux compared to controls

**Figure 4:**
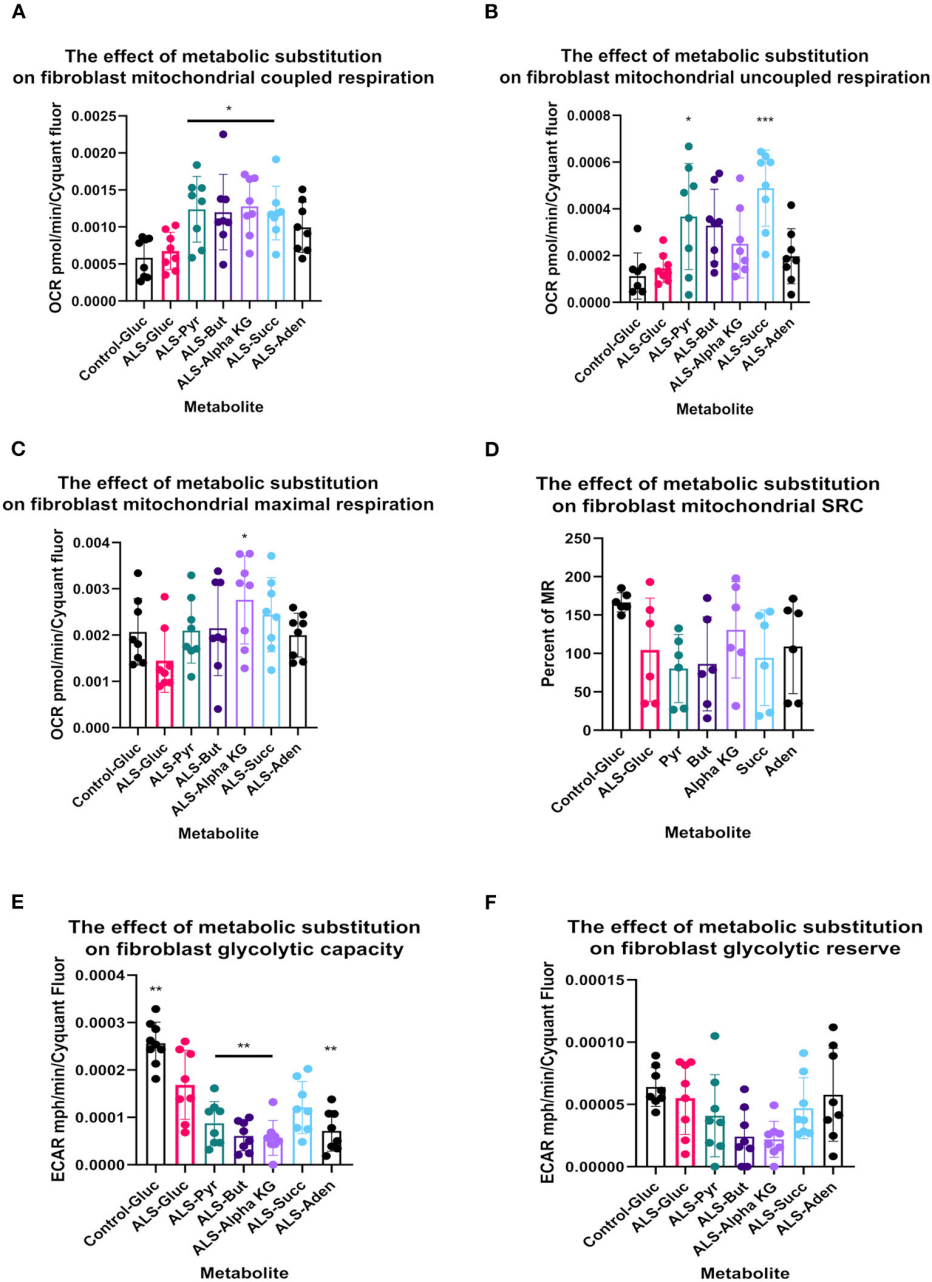
Metabolic substitution reduces glycolytic capacity under stress and promotes mitochondrial respiration in TDP43 fibroblasts. **(A)** The effect of metabolic substitution on fibroblast mitochondrial coupled respiration. **(B)** The effect of metabolic substitution on fibroblast uncoupled respiration. **(C)** The effect of metabolic substitution on fibroblast maximal mitochondrial respiration. **(D)** The effect of metabolic substitution on fibroblast mitochondrial spare respiratory capacity (SRC). **(E)** The effect of metabolic substitution on fibroblast glycolytic capacity. **(F)** The effect of metabolic substitution on fibroblast glycolytic reserve. Data presented as mean with standard deviation of two control and two ALS cases performed n = 3/4 prior to one way ANOVA with Tukey's post-test analysis or Kruskal Wallis non-parametric analysis with Dunn's post-test analysis. *p ≤ 0.05, **p ≤ 0.01. ***p ≤ 0.001.

Metabolic flux analysis on TDP43 fibroblasts confirmed the metabolic profiling and transcriptomic datasets. Under glucose conditions, TDP43 fibroblasts had reduced ECAR, which is a proxy for glycolytic flux and is determined by the hydrogen ions produced by pyruvate to lactate dehydrogenation (Figure 4B). This confirmed the pyruvate metabolism defect observed previously. Under glucose conditions, mitochondrial respiratory (MR) flux was unaffected (Figure 4A). Interestingly, pyruvate substitution (for glucose) worsened the ECAR defect (Figure 4B) whilst concomitantly feeding carbon into the mitochondria (Figure 4A). Pyruvate substitution decreased glycolytic ATP output promoting mitochondrial output similar to targeting the fibroblasts with TCA cycle substrates such as butyrate, succinate and alpha-ketoglutarate, which as expected, increased mitochondrial flux and ATP output both at the expense of glycolytic output (Figures 4A–D) and at the expense of total ATP production. Moreover, adenosine which showed no metabolic defects in TDP43 fibroblasts, mechanistically behaved in a similar manner but unlike pyruvate and the mitochondrial targeted substrates, which all showed defects in the metabolic screen, actually improved total ATP output in TDP43 mutant fibroblasts (Figure 4C).

When we probed mitochondrial and glycolytic flux under conditions of metabolic stress, we found that, across the board, all substrates increased coupled respiratory flux in fibroblasts with the TDP43 truncation but in most cases also increased proton leak, which was not observed with adenosine substitution (Figures 4A, B). This generally led to a reduction in percentage coupling to under 80% in ALS fibroblasts, which under glucose conditions was 81%, compared to 88% in controls (data not shown). Interestingly, although TDP43 truncation had no effect on mitochondrial basal flux, it reduced mitochondrial capacity (MCAP) by approximately 25% (unpaired *t*-test *p* = 0.03), with a similar effect observed on mitochondrial spare respiratory capacity (SRC) (Figures 4C, D). Metabolic substitution recovered MCAP, which is to be expected as MR is increased by metabolic substitution. However, pyruvate and butyrate showed a strong trend for reduced SRC (ANOVA *p* = 0.054, 0.082 respectively), less so for succinate (0.134) as the data were variable. These results suggest that, although TDP43 fibroblasts could use these substrates, it came at the detrimental cost of increased proton leak and reduced mitochondrial SRC, leading to decreased ATP and NADH production.

In terms of glycolytic flux under stress, TDP43 fibroblasts had reduced glycolytic capacity and glycolytic reserve that was worsened by pyruvate, butyrate alpha-KG and succinate (Figures 4E, F). As fibroblasts, like astrocytes, are dependent on glycolysis for their ATP generation this suggested that under times of bioenergetic stress, mobilization of these metabolites into the energy generation pathways was detrimental to the cell.

### Pyruvate metabolism targets are altered in the motor cortex of a TDP43 Y374X case

**Figure 5:**
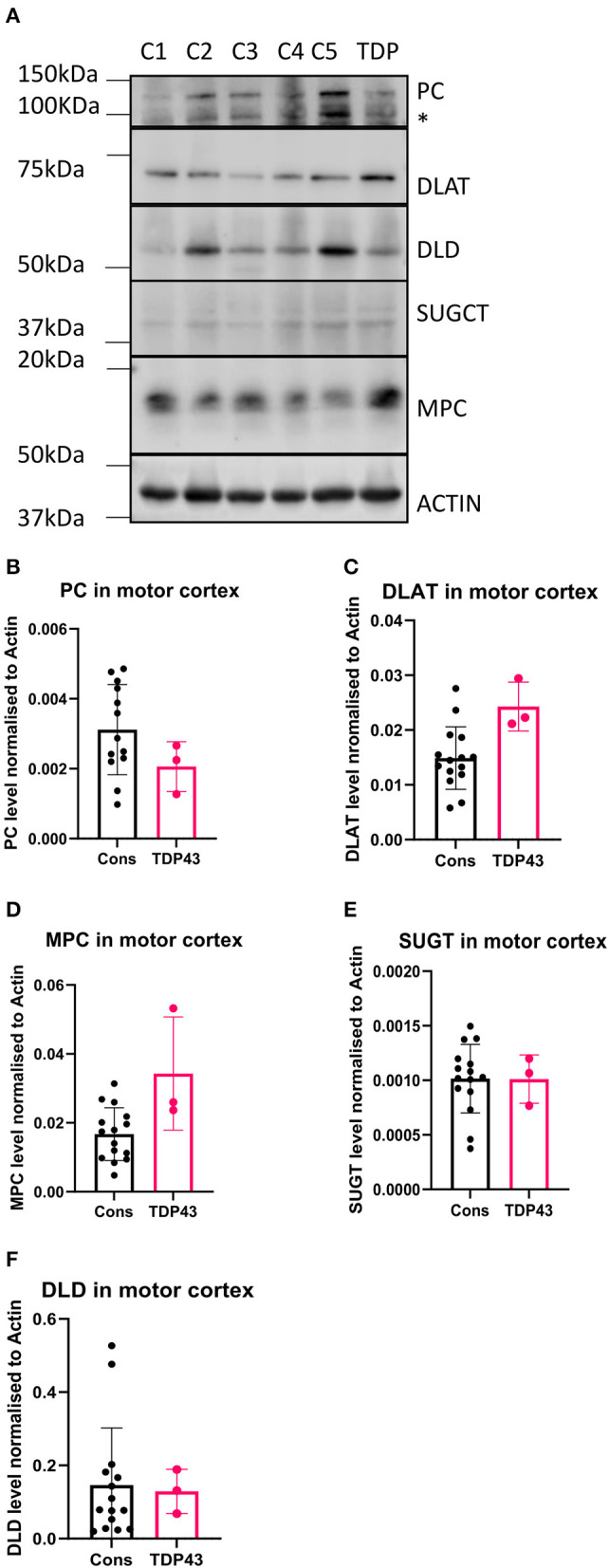
Metabolic markers are altered in a TDP43 truncation case. **(A)** Western blot of protein isolated from 5 control and 1 TDP43 truncation case. **(B)** Densitometry analysis of PC. **(C)** Densitometry analysis of DLAT. **(D)** Densitometry analysis of MPC. **(E)** Densitometry analysis of SUGCT. **(F)** Densitometry analysis of DLD. *Non-specific band. Data presented as mean with standard deviation of 3 technical replicates from 5 control cases and one TDP43 case.

In our previous study we used available motor cortex samples to validate our findings (Cooper-Knock et al., 2022). We repeated this approach in this follow up study to assess whether the metabolomic and transcriptomic findings observed in the fibroblasts isolated from the three TDP43 truncation cases were recapitulated in the one available motor cortex sample from the single available TDP43 truncation case (Case 194). We performed Western blot analysis on six metabolic targets involved in carbon flow in glycolysis and the TCA cycle, namely the mitochondrial pyruvate carrier (MPC2), DLAT the E2 component of pyruvate dehydrogenase, pyruvate carboxylase (PC), DLD, SUGCT and GAA. We could not detect a GAA signal in any of our samples but were able to detect the five other targets (Figure 5A). Interestingly we found alterations in pyruvate metabolism enzymes in the one TDP43 cases, but not exactly in the way we would have expected based on the fibroblast data. In the fibroblast dataset, MPC2 was reduced, whereas in the one TDP43 cortex sample available MPC2 was clearly elevated (Figures 5A, D) indicating alterations in this pathway but distinct to those observed in TDP43 fibroblasts. Furthermore, DLAT and PC enzymes involved in carbon flow into acetyl-CoA and oxaloacetate respectively were altered, with DLAT being increased and PC being decreased. We also measured DLD and SUGCT which were upregulated and downregulated respectively in TDP43 fibroblasts but observed no changes in the TDP43 cortex sample indicating that the changed mechanisms observed may be limited to fibroblasts.

## Conclusions

Our data suggest that TDP43 truncation alters the overall metabolic profile of fibroblasts with specific dysfunction centered around carbon flow into the mitochondria being the primary factor involved. The metabolic intermediate pyruvate is a crucial gateway metabolite in fibroblasts. Enzymes controlling pyruvate metabolism control carbon flow into cytosolic lactate and into the mitochondria via transport through the MPC, prior to dehydrogenation to acetyl-coA or carboxylation to oxaloacetate. The metabolic profiling data suggested that pyruvate metabolism was reduced in TDP43 fibroblasts, the reasons for this may be multifactorial, depending on carbon flow from pyruvate in fibroblasts. Glycolytic flux was reduced under glucose conditions in TDP43 fibroblasts (Figure 3B) suggesting loss of carbon flow into lactate. However, this is not an NADH producing step and therefore the likely cause of loss of pyruvate metabolism observed in the TDP43 fibroblasts was due to entry and metabolism of pyruvate in the mitochondria. This hypothesis was supported by our transcriptomic data which showed a reduction in the gene expression of the mitochondrial pyruvate carrier MPC2. Defects in pyruvate metabolism have previously been observed in C9orf72 fibroblasts (Allen et al., 2019a). Moreover, elevated plasma pyruvate levels have been reported in ALS patients indicating alterations in intracellular metabolism (Lawton et al., 2012) and altered pyruvate metabolism has been observed in cell models of disease (Valbuena et al., 2016). Loss of pyruvate metabolism in the mitochondria would reduce the carbon flow into the TCA cycle. Accordingly, we found alterations in expression of compensatory mitochondrial enzymes including the E3 component of the pyruvate dehydrogenase complex DLD, and ACSS3, which catalyse the synthesis of acetyl-CoA from short-chain fatty acids (Odievre et al., 2005; Yoshimura et al., 2017). Upregulation of both of these enzymes may represent an attempt to maintain acetyl-CoA levels in the TDP43 cases. However, the upregulation of both genes may be multi-factorial in nature as DLD is also a component of the alpha-ketoglutarate dehydrogenase complex and ACSS3 can use butyrate as a substrate. We found significant loss of alpha ketoglutaric acid and butyric acid metabolism in the truncated TDP43 fibroblasts. Therefore, upregulation of these enzymes may also represent an attempt to restore defects in these pathways that feed into the TCA cycle. In addition, we found a defect in the TCA cycle energy substrate succinamic acid the carboxylate anion of succinate. In relation to this, we found loss of the enzyme SUGCT, which catalyses the formation of succinate and glutarate to their respective CoA moieties, with the latter conversion dependent on the presence of succinyl Co-A (Marlaire and Van Schaftingen, 2014). It is not clear whether the loss of succinamic acid metabolism was a consequence of loss of SUGCT or whether alternatively, SUGCT downregulation was due to disruption in succinate metabolism.

Our data suggest a disruption to the carbon flow directed into the TCA cycle caused by TDP43 truncation. Many previous studies have linked TDP43 mutation to mitochondrial dysfunction, for recent reviews see (Jiang and Ngo, 2012; Floare and Allen, 2020; Dafinca et al., 2021). However, there has been limited focus on the TCA cycle as a target for dysfunction in TDP43, even though previous work has shown that TDP43 overexpression in rats inhibits the TCA cycle intermediates succinate and citrate, which was rescued by Parkin (Hebron et al., 2014). Interestingly, recent metabolomic analysis in a TDP43 p.G298S ALS Drosophila model showed general compensatory upregulation of the TCA cycle intermediates in motor neurons including citrate, malate and fumarate (Loganathan et al., 2022). These data somewhat align with our transcriptomic data which show a compensatory upregulation of the mitochondrial genes that would feed carbon into the TCA cycle namely ACSS3 and DLD. Moreover, in the same study, pyruvate supplementation in the TDP43 p.G298S ALS Drosophila model rescued the observed larvae locomotor defects observed by the authors. Our motor cortex analysis also suggested alterations in components on the pyruvate metabolism pathway into the mitochondria (Figure 5) although whether those changes were pathogenic or compensatory remain to be seen and any conclusions are obviously limited because we had only one TDP43 motor cortex sample available from the pedigree with the TARDBP truncating mutation. However, overall these data suggest that promoting carbon flow via pyruvate may be beneficial in the context of TDP43 ALS mutations irrespective of whether they are point or truncation mutations.

Along with the mitochondrial pyruvate carrier, our gene expression analysis identified loss of other mitochondrial membrane transporters including the component of the outer membrane translocase TOMM7 and the inner membrane calcium dependent ATP-Mg/Pi carrier SLC25A23. We have previously postulated that loss of mitochondrial transporters could contribute to the mitochondrial defect observed in ALS, leading to a reduction in metabolites, co-factors and nucleotides in the organelle (Allen et al., 2019a,b). Loss of the pyruvate carrier and SLC25A23 align with this hypothesis and would lead to metabolic and oxidative dysregulation in the mitochondria. However, there seems again to be some compensatory mechanisms occurring in the TDP43 cases, with upregulation of the ATP-Mg/Pi carrier SLC25A24 (Iacobazzi et al., 2017), mitochondrial transcription/translation components and the cytochrome C assembly component COX20. It would be interesting to assess whether this genetic metabolic flexibility is observed in the CNS models of truncated TDP43 (Allen et al., 2019a).

We found in the truncated TDP43 cases, defects in the metabolism of the glucoside salicin, likely caused by loss of alpha-glucosidase (GAA) expression. Reduction in GAA activity has been observed in the spinal cord of the SOD1 mouse model (Dodge et al., 2013). These data indicate a common mechanism of dysfunction between genetic sub-groups of the disease. We also found a defect in the metabolism of the sugar alcohol xylitol, which is used as an artificial sweetener for food supplements. We have previously linked altered xylitol metabolism with age in ALS fibroblasts (Gerou et al., 2021). To the best of our knowledge, this is first study linking defects in xylitol metabolism and TDP43 ALS. Xylitol is metabolized to xylitol-5-phosphate, which via protein phosphatase 2A, activates the transcription factor ChREBP (carbohydrate response element binding protein) (Kawaguchi et al., 2001; Kabashima et al., 2003). ChREBP activates lipogenic genes, including acetyl CoA carboxylase and fatty acid synthase stimulating lipogenesis. Reduction in dietary xylitol metabolism may favor a lipid oxidation rather than a lipid synthesis profile in TDP43 ALS cases.

Finally we found a significant reduction in uridine metabolism in the truncated TDP43 cases. Uridine is a key pyrimidine metabolic intermediate, which has been implicated previously in fibroblasts from sporadic ALS cases (Veyrat-Durebex et al., 2019). Loss of uridine metabolism in truncated TDP43 fibroblasts may be due in part to a reduction of the G-coupled purinergic receptor P2RY13. As P2YRs can be activated by extracellular ATP metabolites including uridine, uridine 5′triphosphate (UTP) and adenine, therefore uridine supplementation may prove beneficial in ALS cases. With this in mind, uridine supplementation in a SOD1 mouse model increased survival in the animal due in part to a reduction of oxidized DNA damage (Amante et al., 2010). As with C9orf72 fibroblasts (Allen et al., 2019a), no significant difference was found in the metabolism of the purine nucleoside inosine (Supplementary Figure 2). However, unlike C9orf72 fibroblasts, no significant defect was observed in the metabolism of purine nucleoside adenosine. This suggests that C9orf72 specific mechanisms rather than TDP43 mutations mechanisms lead to loss of adenosine metabolism.

These data show that truncation of TDP-43 leads to a decrease of the protein in fibroblasts invoking a metabolic phenotype. Loss of TDP43 leads either directly, or indirectly, to defects in relevant pathogenic ALS metabolic pathways. If directly, then that raises the question as to whether the changes are due to loss of the protein or loss of a key C-terminal part of the protein. There have been several “interactome” focused TDP43 papers published that have primarily shown interactions with ribonucleoproteins, RNA binding proteins, ribosomal proteins, splicing factors, that may indirectly effect mitochondrial function due to reduced transcription/translation in a model of TDP43 haploinsufficiency (Freibaum et al., 2010; Ling et al., 2010; Blokhuis et al., 2016). However, within these studies TDP43 has also been shown to interact with mitochondrial proteins involved in energy metabolism including complement component 1q subcomponent binding protein (C1QBP), ATP synthase subunit alpha and the mitochondrial carrier protein SLC25A23, which was significantly reduced in this study. Therefore, TDP43 protein loss may alter the function of these proteins. However, although TDP43 silencing in HELAs has been shown to alter mitochondrial-ER contact sites, disrupting calcium flux, the effect on mitochondrial morphology and the respiratory complex expression was minimal (Peggion et al., 2022).

Therefore, this raises the question as to whether the metabolic changes we observed were due to loss of the C-terminal part of the protein post residue 374? Interestingly, in a recent paper focused on TDP43 with an A382T mutation, isolated fibroblasts displayed fragmented mitochondria, reduced respiration and alterations in levels of TCA cycle and electron transport chain components (Zanini et al., 2022). These included DLD, pyruvate dehydrogenase E1 subunit alpha 1 (PDHA1), the mitochondrial carrier SCL25A4, and many TCA cycle associated enzymes including isocitrate dehydrogenase 1 (IDH1), C1QBP, aconitase 2 (ACO2), citrate synthase (CS), dihydrolipoamide S-succinyltransferase (DLST), fumarate hydratase (FH) and malate dehydrogenase 2 (MDH2). These data align with our study suggesting that the C-terminal region of TDP43 plays an important role in mitochondrial function.

Lastly, it is important not to rule an indirect effect of TDP43 loss/truncation perhaps mediated via STMN2 or UNC13A, which were found to be reduced in the TDP43 truncation cases (Cooper-Knock et al., 2022). STMN2 knockout has recently been shown to lead to damaged mitochondria and microtubule alterations in cultured neurons (Krus et al., 2022; San Juan et al., 2022). Moreover, ^18^F-2-fluoro-2-deoxy-d-glucose-PET imaging of UNC13A rs12608932 variant ALS cases showed hypometabolism is the brain, indicating a metabolic signature in these ALS cases (Calvo et al., 2022). Therefore, evidence is emerging of metabolic phenotypes linked to these genes, which are altered in the presence of TDP-43 proteinopathy.

Irrespective of the mechanisms involved linking TDP43 truncation and the metabolic defects observed, pathophysiological changes in these pathways are found in both sporadic and genetic subtypes of the disease, indicating common pathways with the potential for therapeutic manipulation.

## Data availability statement

The data presented in the study are deposited in the Gene Expression Omnibus (GEO, http://www.ncbi.nlm.nih.gov/geo/) repository, accession number: GSE224138.

## Ethics statement

Informed consent was obtained from all participants before skin sample collection (Study number STH16573, Research Ethics Committee reference 12/YH/0330). Autopsy tissues were donated to the Sheffield Brain Tissue Bank (SBTB) with appropriate consent. The SBTB Management Board gave ethical approval for this study under the provision to act as a Research Tissue Bank as approved by the Scotland A Research Ethics Committee (Ref. 08/MRE00/103).

## Author contributions

SPA, AAS, EKK, ET, KJH, LMC, YHL, SR, NS, JCK, JRH, RJM, GMH, PRH, and JK collected samples, designed experiments, performed experiments, and analyzed data. SPA, RJM, JCK, JRH, GMH, PRH, JK, and PJS provided funding and resources. SPA, AAS, PRH, JK, and PJS wrote the initial manuscript. All authors contributed, edited and revised the manuscript, and approved the submitted version.

## Supplementary material

Supplementary Figure 1 Phenotypic metabolic profiling end point profile of control fibroblasts (black) and TDP43 fibroblasts (orange).

Supplementary Figure 2 TDP43 metabolic screening results.

Supplementary Table 1 All differentially expressed metabolic genes between control and truncated TDP43 cases.
