# Supplementary figures and images for: A Y374X TDP43 truncation leads to an altered metabolic profile in amyotrophic lateral sclerosis fibroblasts driven by pyruvate and TCA cycle intermediate alterations
Source: Front Aging Neurosci. 2023 May 11;15:1151848. doi: 10.3389/fnagi.2023.1151848 (PMC10213779; doi:10.3389/fnagi.2023.1151848)

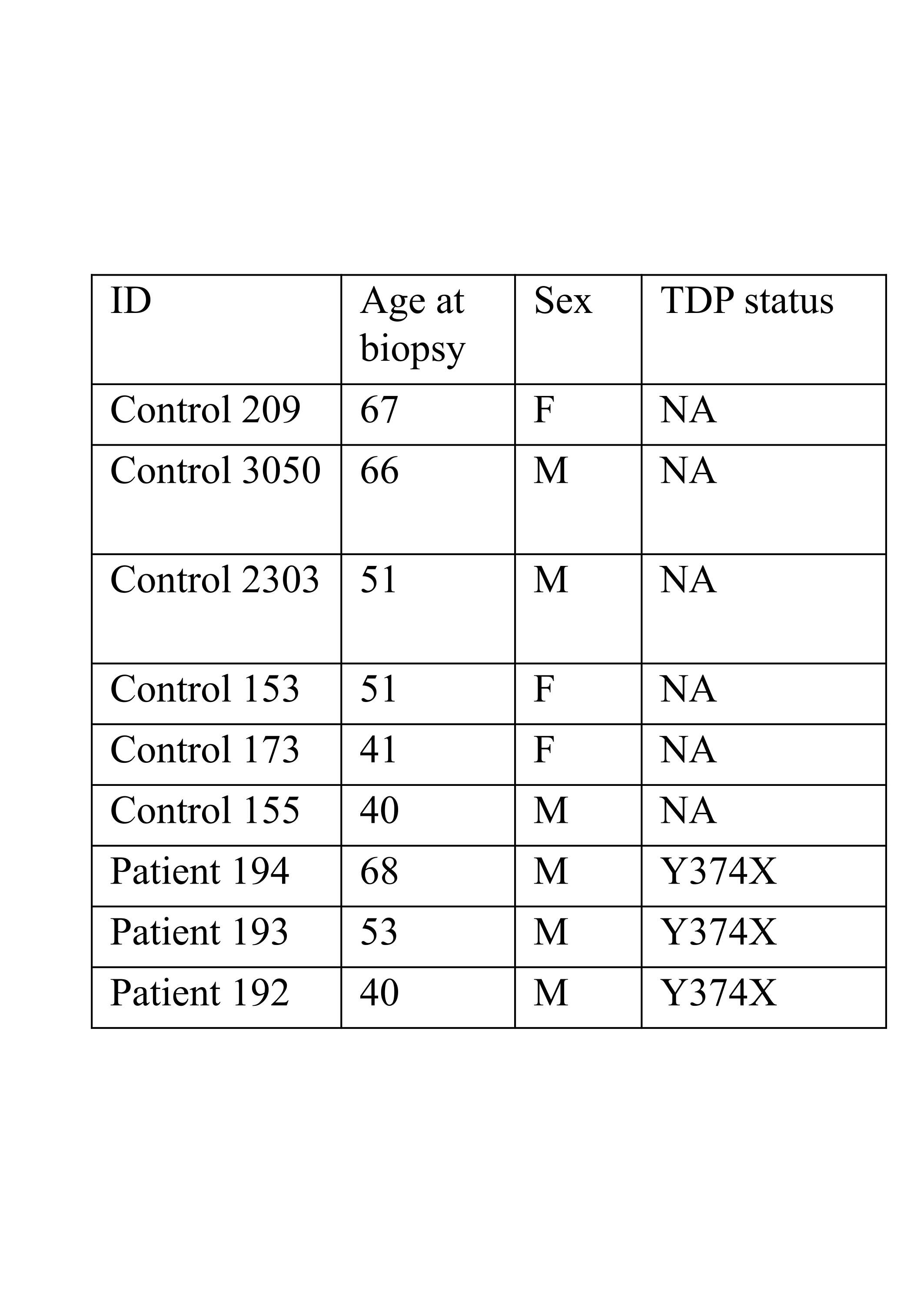

Supplement: Supplementary file 1 [file Image_1.TIF]

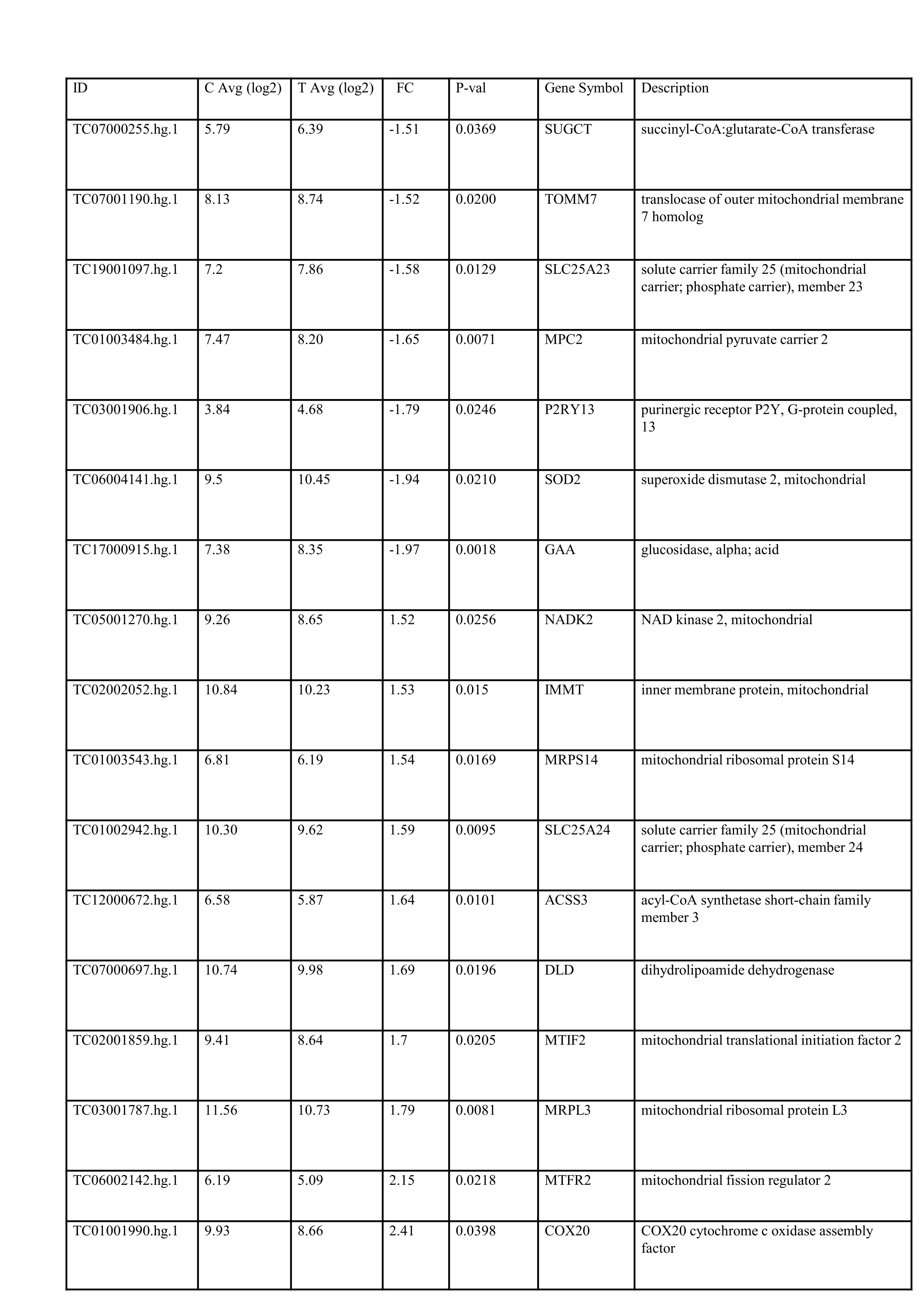

Supplement: Supplementary Figure 1 — Phenotypic metabolic profiling end point profile of control fibroblasts (black) and TDP43 fibroblasts (orange). [file Image_2.TIF]

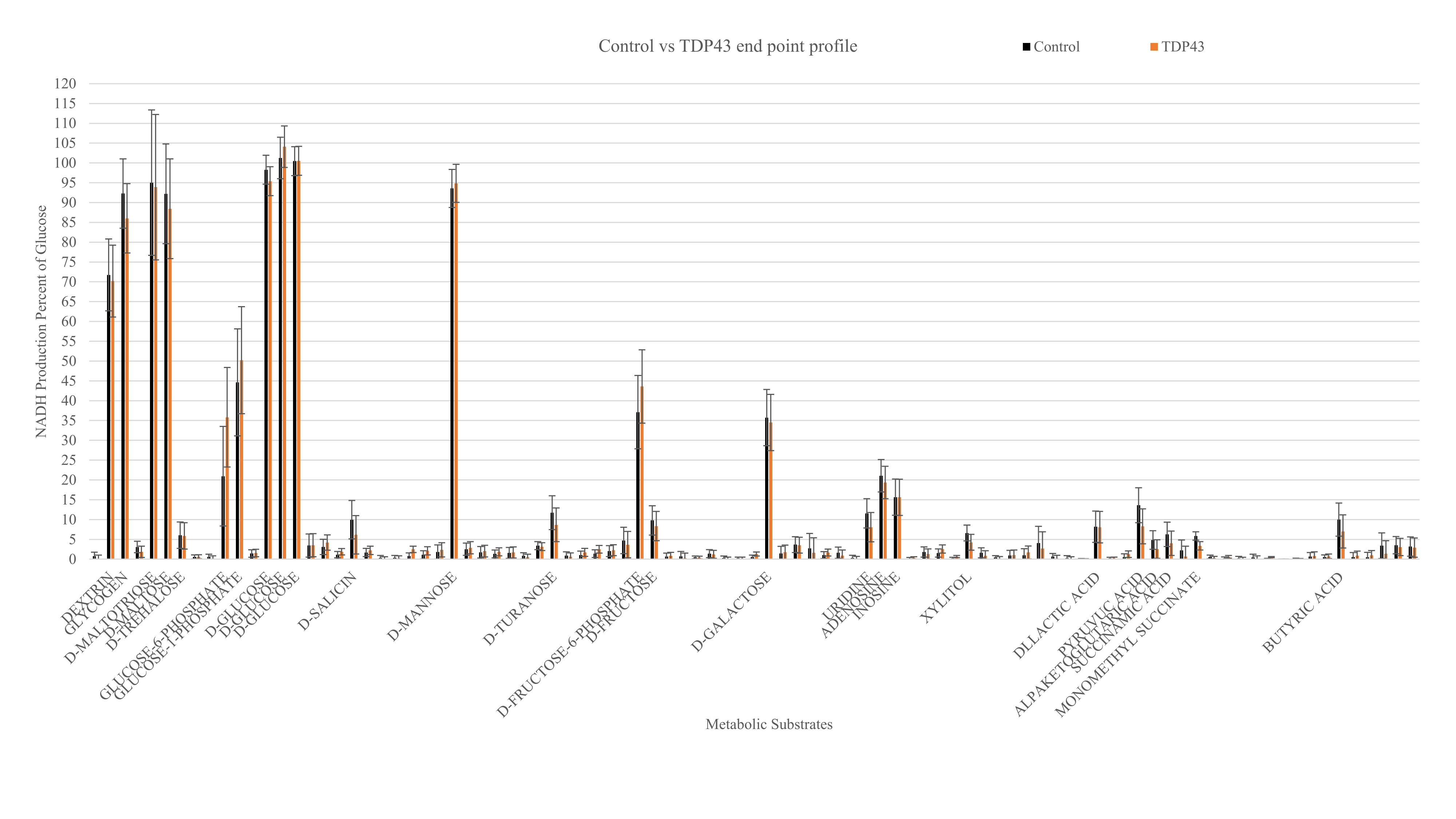

Supplement: Supplementary file 3 [file Image_3.TIF]

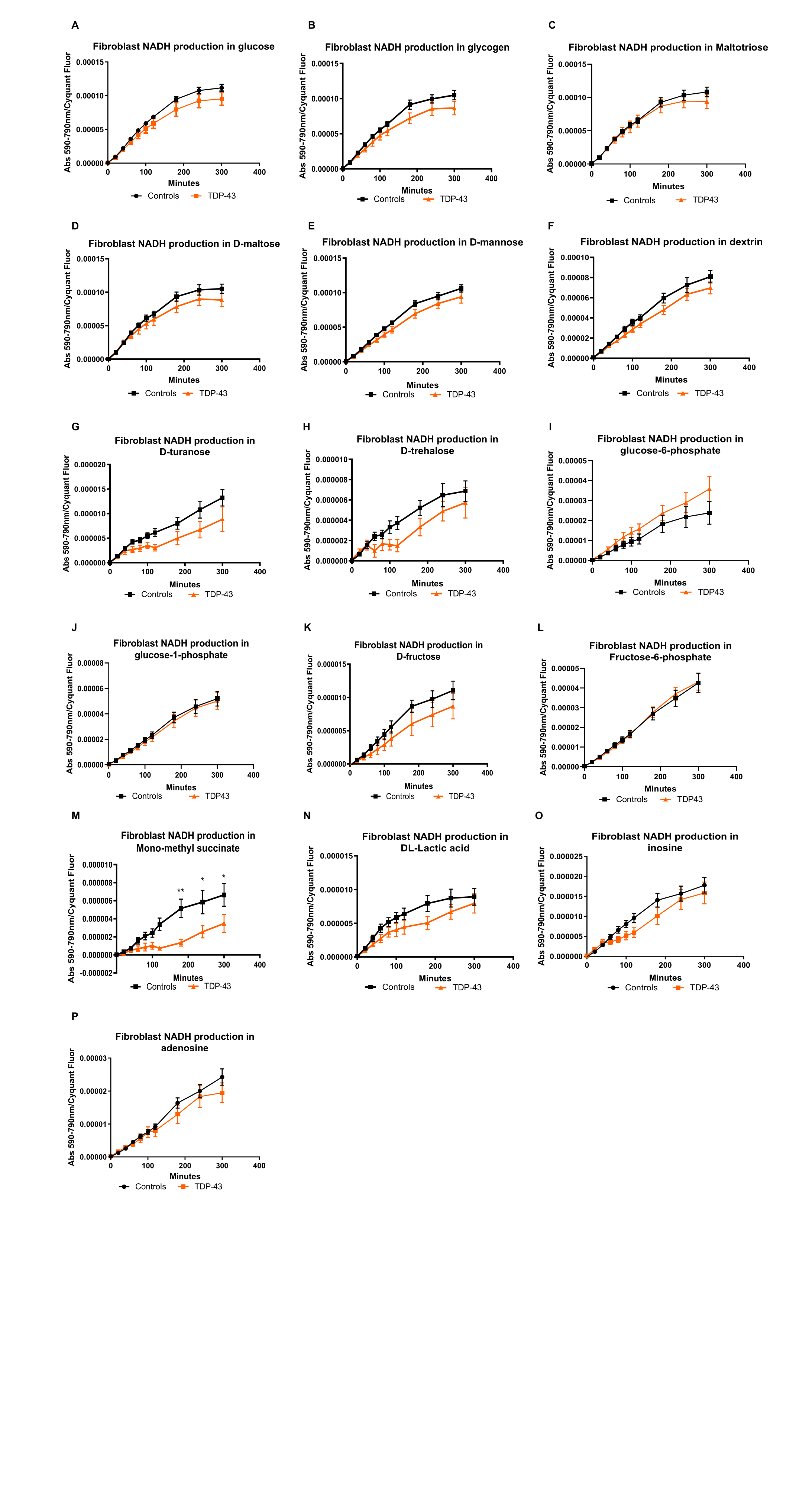

Supplement: Supplementary Figure 2 — TDP43 metabolic screening results. [file Image_4.TIF]
